# Single nucleotide polymorphisms in *MLH1* predict poor prognosis of hepatocellular carcinoma in a Chinese population

**DOI:** 10.18632/oncotarget.16899

**Published:** 2017-04-06

**Authors:** Xiaonian Zhu, Wei Liu, Xiaoqiang Qiu, Zhigang Wang, Chao Tan, Chunhua Bei, Linyuan Qin, Yuan Ren, Shengkui Tan

**Affiliations:** ^1^ Department of Epidemiology and Statistics, School of Public Health, Guilin Medical University, Guilin 541004, Guangxi, People’s Republic of China; ^2^ Department of Epidemiology, School of Public Health, Guangxi Medical University, Nanning 530021, Guangxi, People’s Republic of China

**Keywords:** MLH1, HCC, SNP, risk, prognosis

## Abstract

Hepatocellular carcinoma (HCC) is a malignant cancer causing deleterious health effect worldwide, especially in China. So far clinical cure rate and long-term survival rate of HCC remains low. Most HCC patients after cancer resection have recurrence or metastasis within 5 years. This study aims to explore the genetic association of *mutL homolog 1* (*MLH1*) polymorphisms with HCC risk and prognosis. Four candidate *MLH1* polymorphisms, rs1800734, rs10849, rs3774343 and rs1540354 were studied from a hospital-based case-control study including 1,036 cases (HCC patients) and 1,036 controls (non-HCC patients) in Guangxi, China. All these SNPs interacted with environmental risk factors, such as HBV infection, alcohol intake and smoking in the pathogenesis of HCC. However, only rs1800734 had significant difference between cases and controls. Compared to the AA genotype, patients with AG, GG and AG/GG genotype of rs1800734 had an increased risk of HCC [ORs (95% CI) = 1.217 (1.074∼1.536), 1.745 (1.301∼2.591) and 1.291 (1.126∼1.687)] and a decreased survival time [co-dominant, HR (95% CI) = 1.553 (1.257∼1.920); dominant, HR (95% CI) = 2.207 (1.572∼3.100)]. Furthermore, we found that tumor number, tumor staging, metastasis and rs1800734 were associated with the overall survival of HCC patients by multivariate COX regression analysis. No significant difference was found between the other three *MLH1* polymorphisms with HCC risk and prognosis. Our study suggests *MLH1* SNP, rs1800734 as a new predictor for poor prognosis of HCC patients.

## INTRODUCTION

Hepatocellular carcinoma (HCC) is a common malignant tumor of digestive system, and a complex disease caused by interactions between multiple genetic and environmental factors [1, 2]. There are about 800,000 new cases of HCC around the world each year, of which more than 50% occurs in China [3, 4]. Guangxi is one region with the highest incidence of HCC in China, where HCC is the leading cause of tumor-related mortality. Because of insidious onset and high malignancy, most HCC patients are diagnosed in advanced stage with poor prognosis. Although comprehensive treatment based on surgery has improved the curative effect of HCC greatly, clinical cure rate and long-term survival rate have no significant improvement. Recently, studies on the causes, prognosis markers, molecular targets and carcinogenesis mechanisms of HCC are increasing widely [5–8], but the exact molecular mechanism is still not well understood. Therefore, it is very crucial to find the molecular markers which can be used in the risk and prognosis evaluation for the prevention and treatment of HCC.

Single nucleotide polymorphism (SNP) is the third generation of molecular marker and one of the most common genetic variations in human. Studies show that SNP can not only be used as a genetic marker locating disease gene, some SNP itself can also directly lead to the occurrence of diseases [9, 10]. Thus SNP has crucial function and application in disease risk assessment, early diagnosis, prevention, treatment and drug development [11–13]. Mismatch repair (MMR) system is a DNA repair system with a high fidelity. It mainly repairs base mismatch and insertion/deletion ring produced in the synthesis of DNA to maintain the stability of the whole genome [14–16]. MutL homolog 1 (MLH1) plays a key role in the MMR system. MLH1 recognizes and repairs the mismatched bases in the process of DNA replication, and also recruits other mismatch repair proteins to the mismatch site to correct DNA replication error [17, 18]. Studies found that SNPs of specific DNA repair genes could affect the expression level and activity of enzymes and individual DNA damage repair efficiency. Repair gene defects may lead to genetic instability and cancer occurrence, suggesting that individual differences in cancer risk was related to polymorphisms of specific repair genes [19–21]. It has been reported *MLH1* polymorphisms had a correlation with the pathogenesis of colorectal cancer, lung cancer, breast cancer, prostate cancer, head and neck squamous cell carcinoma, oral squamous cell carcinoma [19–27]. However, there is only a few researches about the relationship between *MLH1* polymorphism and HCC [28], and the results still need further verification due to race, nation, region and individual differences.

In order to clarify genetic variations of *MLH1* in HCC, this study aims to screen potential *MLH1* SNPs in a case-control study from a HCC population in Guangxi. Four *MLH1* SNPs, rs1800734, rs10849, rs3774343 and rs1540354, that minor allele frequency (MAF) was more than 0.05, were selected from the National Institute of Environmental Health Sciences (NIEHS) database. Through the investigation and collection of demographic and clinical information from the study population, the relationships between these *MLH1* SNPs with risk and prognosis of HCC were analyzed. Finally, we found that rs1800734 was a new predictor for poor prognosis of HCC patients.

## RESULTS

### General demographic characteristics of study population and Hardy-Weinberg equilibrium test results

The general demographic characteristics of study population were shown in Table 1. The case and control group were significantly different at alcohol intake, smoking, HBV infection and family history (*P* < 0.001). However, there was no difference about gender, age or nation between the two groups (*P* > 0.05).

**Table 1:** Distributions of general demographic characteristics and environmental risk factors in the controls and cases

Characteristics	Controls (*n* = 1036)	Cases (*n* = 1036)	χ^2^	*P*
Age (years)				
< 50	497	532	2.365	0.124
≥ 50	539	504		
Gender				
Male	896	896	0.000	1.000
Female	140	140		
Nation				
Han	736	715	1.048	0.592
Zhuang	286	305		
Others	14	16		
Smoking				
No	879	674	108.03	**< 0.001**
Yes	157	362		
Alcohol intake				
No	895	689	113.75	**< 0.001**
Yes	141	347		
HBV infection				
No	953	176	1174.97	**< 0.001**
Yes	83	860		
Family history				
No	1022	975	30.56	**< 0.001**
Yes	14	61		

Bold values indicate significance.

Haploview 4.2 test showed genotype distribution of the four *MLH1* SNPs, rs1800734, rs10849, rs3774343, and rs1540354 accorded with the HWE equilibrium in the control group (Supplementary Table 1).

### The relationship between genotype distribution of *MLH1* SNPs and HCC risk

Multivariate logistic regression analysis showed that *MLH1* SNP, rs1800734 had a significant difference between the case and control group (Table 2, *P* < 0.05). After adjusted for age, gender, smoking, alcohol intake and HBV infection, compared with the AA genotype, the ORs (95% CI) of AG, GG and AG/GG genotype with HCC risk were 1.217 (1.074∼1.536), 1.745 (1.301∼2.591) and 1.291 (1.126∼1.687), respectively. The other three *MLH1* SNPs, rs10849, rs3774343 and rs1540354 had no statistically significant differences between the case and control group (*P* > 0.05).

**Table 2:** The genotype frequencies of MLH1 polymorphisms and HCC risk

Genotypes	Cases (%)	Controls (%)	Frequencies in HapMap project (%)	OR (95% CI)^a^	OR (95% CI)^b^
**rs1800734**					
AA	393 (37.16)	338 (32.63)	30.23	1.000	1.000
AG	522 (50.39)	529 (51.06)	44.19	1.178 (0.975∼1.424)	**1.217 (1.074∼1.536)**
GG	121 (12.45)	169 (16.31)	25.58	**1.624 (1.234∼2.138)**	**1.745 (1.301∼2.591)**
AG/GG	643 (62.84)	675 (67.37)	69.77	**1.221 (1.018∼1.463)**	**1.291 (1.126∼1.687)**
A	1308 (63.13)	1205 (58.16)	52.33	-	-
G	764 (36.87)	867 (41.84)	47.67	-	-
**rs10849**					
AA	7 (0.67)	3 (0.29)	0	1.000	1.000
AG	151 (14.58)	162 (15.64)	0.19	2.503 (0.636∼9.857)	2.625 (0.651∼11.236)
GG	878 (84.75)	871 (84.07)	0.81	2.315 (0.597∼8.890)	2.431 (0.605∼9.873)
AG/GG	1029 (99.33)	1033 (99.71)	100.00	2.342 (0.604∼9.083)	2.526 (0.612∼10.585)
A	165 (7.96)	168 (8.11)	9.30	-	-
G	1907 (92.04)	1904 (91.89)	90.70	-	-
**rs3774343**					
CC	8 (0.77)	2 (0.19)	0	1.000	1.000
CT	143 (13.80)	125 (12.06)	11.63	3.497 (0.729∼16.772)	3.624 (0.756∼19.245)
TT	885 (85.43)	909 (87.75)	88.37	4.108 (0.870∼19.401)	4.162 (0.923∼21.523)
CT/TT	1028 (90.23)	1034 (99.81)	100.00	4.023 (0.852∼18.992)	4.101 (0.891∼20.317)
C	159 (7.67)	129 (6.23)	5.81	-	-
T	1913 (92.33)	1943 (93.77)	94.19	-	-
**rs1540354**					
AA	96 (9.27)	81 (7.82)	13.95	1.000	1.000
AT	489 (47.20)	447 (43.15)	39.53	1.083 (0.785∼1.495)	1.125 (0.813∼1.542)
TT	451 (43.53)	508 (49.03)	46.51	1.335 (0.968∼1.842)	1.363 (0.971∼1.876)
AT/TT	940 (90.73)	955 (92.18)	86.04	1.204 (0.884∼1.640)	1.237 (0.896∼1.679)
A	681 (32.87)	609 (29.39)	33.72	-	-
T	1391 (67.13)	1463 (70.61)	66.28	-	-

a: OR (95% CI) not adjusted; b: OR (95% CI) adjusted by logistic regression for age, gender, nation, smoking, alcohol intake, HBV infection, and HCC family history. Bold values indicate significance.

### Gene-environment and SNP-SNP interaction

Logistic regression model analysis showed that rs1800734, rs10849 and rs3774343 had interactions with such environment factors, HBV infection, alcohol intake and smoking in the pathogenesis of HCC (Table 3, *P* < 0.05).

**Table 3:** Gene-environment interaction

Factors	*β*	*S.E.*	*Wald χ*^*2*^	OR (95% CI)^a^	*P*
rs1800734 × Smoking	0.263	0.116	6.354	1.195(1.040∼2.270)	**0.012**
rs1800734 × Alcohol intake	0.358	0.103	14.660	1.294(1.134∼2.132)	**0.000**
rs1800734 × HBV infection	2.132	0.127	735.759	8.685(6.136∼13.246)	**0.000**
rs1800734 × Family history	0.210	0.235	1.862	1.105(0.748∼1.982)	0.176
rs10849 × Smoking	0.253	0.109	6.102	1.183(1.036∼2.131)	**0.014**
rs10849 × Alcohol intake	0.342	0.121	11.638	1.217(1.097∼2.061)	**0.000**
rs10849 × HBV infection	1.532	0.127	657.842	5.685(4.136∼9.246)	**0.000**
rs10849 × Family history	0.198	0.213	1.721	1.079(0.676∼1.837)	0.225
rs3774343 × Smoking	0.232	0.123	5.657	1.152(1.027∼1.846)	**0.021**
rs3774343 × Alcohol intake	0.326	0.113	9.597	1.198(1.071∼1.956)	**0.007**
rs3774343 × HBV infection	1.392	0.115	548.985	4.913(3.694∼8.635)	**0.000**
rs3774343 × Family history	0.191	0.261	1.678	1.024(0.651∼1.736)	0.292
rs1540354 × Smoking	0.245	0.129	6.047	1.197(1.065∼1.976)	**0.019**
rs1540354 × Alcohol intake	0.317	0.124	10.436	1.209(1.087∼2.012)	**0.011**
rs1540354 × HBV infection	1.516	0.117	632.761	5.215(3.956∼8.957)	**0.000**
rs1540354 × Family history	0.211	0.221	1.702	1.105(0.672∼1.894)	0.258

^a^: OR (95% CI) adjusted by logistic regression for age, gender, nations, smoking, alcohol intake, HBV infection, and HCC family history. Bold values indicate significance.

In addition, as shown in Supplementary Table 2, rs1800734 and rs10849, rs1800734 and rs3774343, rs1800734 and rs1540354 had SNP-SNP interactions in the pathogenesis of HCC (*P* < 0.05), and these interactions could increase HCC risk.

### Associations between *MLH1* polymorphisms with clinical-pathological characteristics and the prognosis of HCC patients

As shown in Table 4, *MLH1* SNP, rs1800734 was correlated with tumor size, staging and AFP level of HCC patients (*P* < 0.05), while rs10849, rs3774343 and rs1540354 had no association with these clinical-pathological characteristics of HCC patients.

**Table 4:** The associations between the MLH1 polymorphisms and clinical characteristics of HCC patients

Variables	AA		AG/GG	OR (95% CI)^a^	OR (95% CI)^b^
**rs1800734**					
Tumor size					
< 5 cm	311 (29.17)	455 (70.83)		1.000	1.000
≥ 5 cm	82 (34.56)	188 (63.44)		1.567 (1.165∼2.109)	1.671 (1.215∼2.252)
Tumor number					
solitary	332 (30.58)	514 (69.42)		1.000	1.000
multiple	61 (29.91)	129 (70.09)		1.366 (0.978∼1.908)	1.379 (0.988∼1.926)
TNM staging					
T1 + T2	337 (31.30)	518 (68.70)		1.000	1.000
T3 + T4	56 (26.14)	125 (74.86)		1.452 (1.030∼2.048)	1.545 (1.092∼2.185)
AFP level (ng/ml)					
< 400	152 (37.16)	206 (62.84)		1.000	1.000
≥ 400	241 (29.45)	437 (70.55)		1.338 (1.030∼1.739)	1.463 (1.126∼1.903)
Lymphatic metastasis					
No	343 (29.02)	535 (70.98)		1.000	1.000
Yes	50 (40.15)	107 (59.85)		1.372 (0.955∼1.971)	1.433 (0.996∼2.061)
**rs10849**					
Tumor size					
< 5 cm	5 (4.64)	761 (95.36)		1.000	1.000
≥ 5 cm	2 (7.32)	268 (92.68)		0.880 (0.170∼4.565)	0.913 (0.215∼5.294)
Tumor number					
solitary	6 (5.50)	840 (94.50)		1.000	1.000
multiple	1 (2.56)	189 (97.44)		1.350 (0.162∼11.279)	1.401 (0.168∼11.706)
TNM staging					
T1+T2	6 (5.73)	849 (94.27)		1.000	1.000
T3+T4	1 (1.96)	180 (98.04)		1.272 (0.152∼10.631)	1.315 (0.163∼11.129)
AFP level (ng/ml)					
< 400	3 (6.89)	355 (93.10)		1.000	1.000
≥ 400	4 (4.74)	674 (95.26)		1.424 (0.317∼6.397)	1.87 (0.331∼6.681)
Lymphatic metastasis					
No	7 (5.24)	871 (94.76)		1.000	1.000
Yes	0 (38.22)	157 (61.78)		0.000	0.000

**Variables**	**CC**	**CT/TT**		**OR (95% CI)^a^**	**OR (95% CI)^b^**
**rs3774343**					
Tumor size					
< 5 cm	6 (3.23)	760 (96.77)		1.000	1.000
≥ 5 cm	2 (2.44)	268 (97.56)		1.058 (0.212∼5.273)	1.117 (0.224∼5.569)
Tumor number					
solitary	7 (3.09)	839 (96.91)		1.000	1.000
multiple	1 (2.56)	189 (97.44)		1.577 (0.193∼12.893)	1.635 (0.206∼13.614)
TNM staging					
T1 + T2	7 (3.58)	848 (96.42)		1.000	1.000
T3 + T4	1 (3.92)	180 (96.08)		1.486 (0.182∼12.151)	1.537 (0.196∼13.513)
AFP level (ng/ml)					
< 400	3 (1.02)	355 (98.98)		1.000	1.000
≥ 400	5 (3.88)	673 (96.12)		1.137 (0.270∼4.787)	1.198 (0.305∼5.477)
Lymphatic metastasis					
No	8 (3.50)	870 (96.50)		1.000	1.000
Yes	0 (4.55)	157 (95.45)		0.000	0.000

**Variables**	**AA**	**AT/TT**		**OR (95% CI)^a^**	**OR (95% CI)^b^**
**rs1540354**					
Tumor size					
< 5 cm	75 (67.34)	691 (32.66)		1.000	1.000
≥ 5 cm	21 (67.07)	249 (32.93)		1.287 (0.777∼2.133)	1.369 (0.819∼2.288)
Tumor number					
solitary	81 (69.76)	765 (30.24)		1.000	1.000
multiple	15 (66.66)	175 (33.34)		1.235 (0.695∼2.195)	1.324 (0.733∼2.389)
TNM staging					
T1 + T2	77 (70.25)	778 (29.75)		1.000	1.000
T3 + T4	19 (64.00)	162 (36.00)		0.844 (0.497∼1.433)	0.875 (0.518∼1.532)
AFP level (ng/ml)					
< 400	36 (69.39)	322 (30.61)		1.000	1.000
≥ 400	60 (69.40)	618 (30.60)		1.152 (0.746∼1.778)	1.216 (0.792∼1.865)
Lymphatic metastasis					
No	86 (68.31)	792 (31.69)		1.000	1.000
Yes	10 (75.00)	148 (25.00)		1.607 (0.816∼3.166)	1.786 (0.879∼3.627)

^a^: OR (95% CI) not adjusted; ^b^: OR (95% CI) adjusted by logistic regression for age, gender, nation, smoking, alcohol intake, HBV infection, and HCC family history.

At the end of the follow-up, there were 37.84% (165/436) of HCC patients died. We found that the prognosis of HCC had a correlation with these clinical-pathological characteristics of patients, such as tumor size, number, staging, AFP level and lymph node metastasis (*P* < 0.05, Table 5), but was not associated with age, gender, HBV infection or family history (*P* > 0.05).

**Table 5:** The associations between general demographic and clinical characteristics with the prognosis of HCC patients

Variables	Cases	Survivors	MST	Log-rank *P*	HR (95% CI)
	*N* = 436	*N* = 271	Months		
Age (years)					
< 50	268	172	49.0	0.650	1.000
≥ 50	168	99	46.7		1.074 (0.788–1.464)
Gender					
Male	353	222	48.7	0.723	1.000
Female	83	49	46.4		0.934 (0.639–1.365)
HBV infection					
No	62	38	46.4	0.951	1.000
Yes	374	233	48.7		0.821 (0.617–1.467)
Family history					
No	399	248	48.6	0.917	1.000
Yes	37	23	46.7		0.971 (0.561–1.681)
Tumor size					
< 5 cm	243	171	51.5	**0.001**	1.000
≥ 5 cm	193	100	44.9		1.668 (1.226–2.270)
Tumor number					
solitary	288	202	51.5	**0.000**	1.000
multiple	148	69	43.5		1.746 (1.300–2.395)
TNM staging					
T1 + T2	300	214	56.0	**0.000**	1.000
T3 + T4	136	57	38.4		1.471 (1.053–2.054)
AFP level (ng/ml)					
< 400	169	120	51.0	**0.023**	1.000
≥ 400	267	151	45.3		1.520 (1.080–2.138)
Lymphatic metastasis					
No	381	254	49.6	**0.000**	1.000
Yes	55	17	32.3		2.458 (1.710–3.533)

Bold values indicate significance.

**Figure 1:**
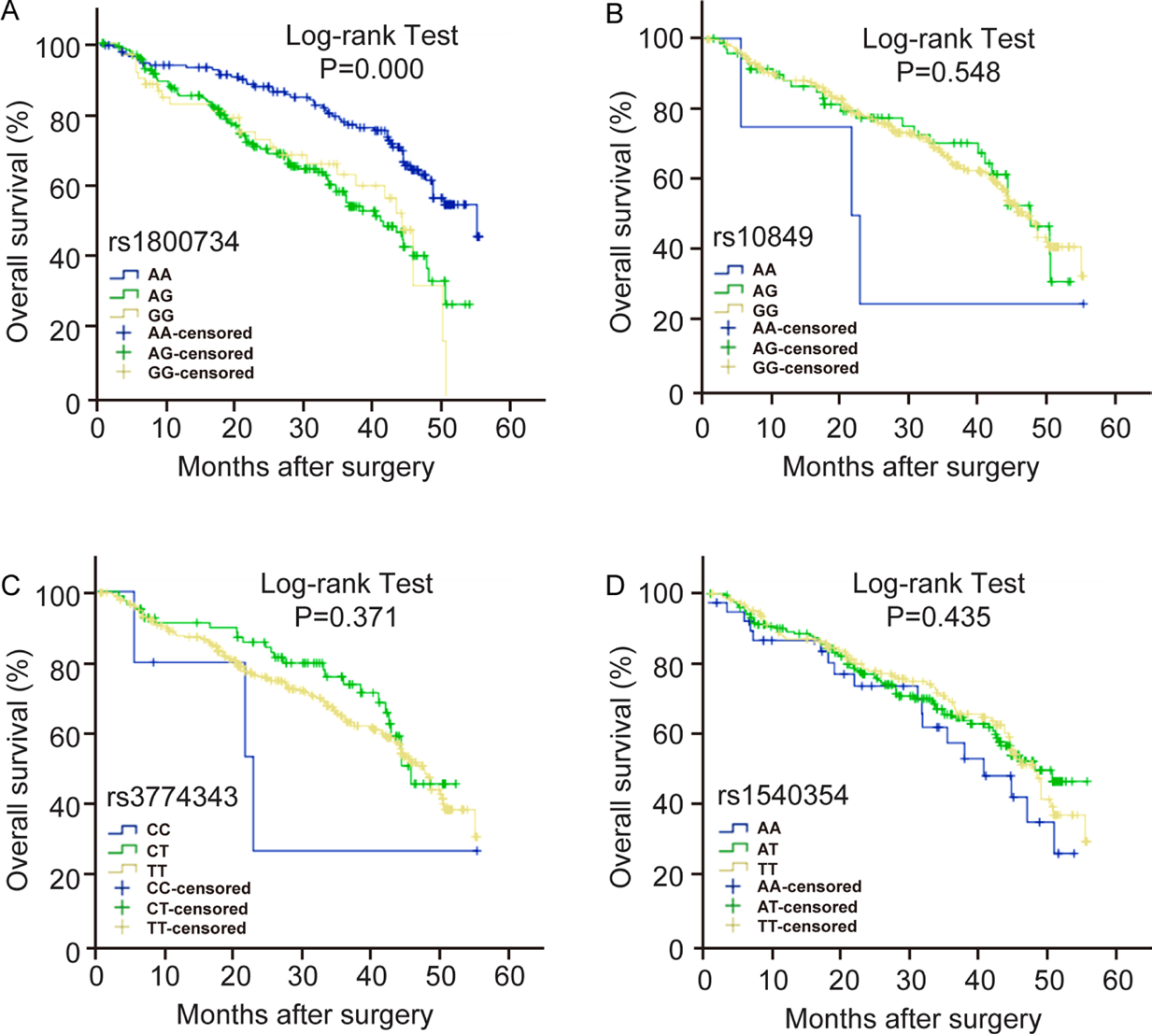
The effect of *MLH1* polymorphisms on the prognosis of HCC patients Kaplan-Meier overall survival curves for HCC patients based on co-dominate genotypes of (**A**) rs1800734, (**B**) rs10849, (**C**) rs3774343, (**D**) rs1540354. *P* value is from the log-rank test.

Moreover, the co-dominant and dominant models of rs1800734 had a significant influence on the prognosis of HCC (Table 6, Figure 1 and Supplementary Figure 1). Compared with the AA genotype, the survival time of HCC patients with AG, GG and AG/GG genotype significantly decreased (*P* < 0.05). But the recessive model of rs1800734 had no effect on the survival time of HCC patients. The genotypes of the other three *MLH1* SNPs, rs10849, rs3774343 and rs1540354 had no effect on the survival time of HCC patients (Figure 1 and Supplementary Figure 1).

**Table 6:** The associations between MLH1 polymorphisms and the prognosis of HCC patients

SNPs	Genotypes	Survivors / Cases	MST (Months)	Log-rank *P*	HR (95% CI)
		(*N* = 271) / (*N* = 436)			
**rs1800734**					
Co-dominant	AA	110/163	56.0	**0.000**	1.553 (1.257–1.920)
	AG	126/212	42.0		
	GG	35/61	45.1		
Dominant	AA	110/163	56.0	**0.000**	2.207 (1.572–3.100)
	AG/GG	161/273	44.3		
Recessive	AG/AA	236/375	48.7	0.120	1.393 (0.915–2.122)
	GG	35/61	45.1		
**rs10849**					
Co-dominant	AA	1/4	22.6	0.548	0.908 (0.631–1.307)
	AG	47/72	48.6		
	GG	223/360	47.6		
Dominant	AA	1/4	22.6	0.273	0.530 (0.166–1.685)
	AG/GG	270/432	48.6		
					
Recessive	AG/AA	48/76	48.6	0.780	0.944 (0.628–1.418)
	GG	223/360	47.6		
**rs3774343**					
Co-dominant	CC	2/5	23.8	0.371	1.091 (0.756–1.573)
	CT	55/81	46.7		
	TT	214/350	48.6		
					
Dominant	CC	2/5	23.8	0.316	0.559 (0.176–1.776)
	CT/TT	269/431	48.6		
					
Recessive	CT/CC	57/86	46.7	0.450	1.167 (0.781–1.744)
	TT	214/350	48.6		
**rs1540354**					
Co-dominant	AA	21/39	41.3	0.435	0.903 (0.711–1.148)
	AT	133/208	51.2		
	TT	117/189	48.6		
Dominant	AA	21/39	41.3	0.197	0.726 (0.445–1.184)
	AT/TT	250/397	48.7		
Recessive	AT/AA	154/247	47.6	0.733	0.948 (0.696–1.290)
	TT	117/189	48.6		

Bold values indicate significance.

### Regression analysis for the survival of HCC patients

We conducted a multivariate regression analysis combined *MLH1* polymorphisms, demographic and clinical-pathological characteristics of HCC patients. As shown in Table 7, these factors, such as rs1800734, tumor number, tumor staging and metastasis, were associated with the death risk of HCC patients.

**Table 7:** COX regression analysis of the prognosis of HCC patients

Variables	*β*	S.E.	HR	95% CI	*P*
Tumor number	0.496	0.164	1.642	1.191–2.264	**0.002**
TNM staging	0.691	0.162	1.995	1.452–2.742	**0.000**
Metastasis	1.184	0.204	3.268	2.191–74.875	**0.000**
rs1800734	1.289	0.194	3.629	2.481–5.310	**0.000**

Bold values indicate significance.

## DISCUSSION

As the most common malignant tumor in southern Guangxi, HCC is a complex process that multiple genes and environmental factors involved [1, 2]. It is well known that environmental carcinogens cause DNA damage by continuing to attack the genomic DNA. If the damage DNA can’t be repaired timely and effectively, the accumulated damage will lead to an increased genomic instability, resulting in cell apoptosis, deregulated cell proliferation and differentiation, eventually cancer [17, 29, 30]. Recent studies report that MLH1, a mismatch repair gene, plays an important role in carcinogenesis [19, 27, 31, 32]. SNPs of mismatch repair genes are thought to provide useful information for tumor diagnosis, suggesting that SNPs of *MLH1* may have potential value for diagnosis of HCC. However, the relationship between *MLH1* polymorphisms and HCC has not been reported in Chinese population yet.

This study analyzed the relationship between *MLH1* polymorphisms and HCC susceptibility in Guangxi area, where there is a high incidence of HCC every year. Finally, we found that the genotype distribution of rs1800734 had a significant difference between cases and controls. The AG, GG and AG/GG genotype of rs1800734 increased HCC risk compared with the AA genotype. But the other three SNPs of *MLH1*, rs10849, rs3774343 and rs1540354 were not correlated with HCC risk.

It has been reported that different populations have different genotype distribution of *MLH1* polymorphisms [15]. The genotype of *MLH1* polymorphisms in European and American population was mainly GG, while our results were in accordance with the genotype in Asians [24, 28, 33]. LO et al. explored the relationship between *MLH1* polymorphisms and lung cancer risk in a case-control study and discovered that rs1800734 was closely related to the occurrence of lung cancer [24]. GG genotype of rs1800734 increased the lung cancer risk compared to AA genotype. Due to genetic differences between different regions and populations, we found only rs1800734 had a correlation with the occurrence and development of HCC. The result is consistent with previous study that rs1800734 significantly increased HCC risk [28]. Whether the other *MLH1* polymorphisms are associated with HCC needs a prospective and large sample study to be verified.

Previous studies indicated that HBV infection, smoking, alcohol intake and family history of cancer were important environmental risk factors of HCC [6, 34, 35]. Thus we conducted gene-environment interaction analysis between the four *MLH1* polymorphisms and these environmental risk factors. The four *MLH1* polymorphisms were found to interact with HBV infection, alcohol intake and smoking, and increase the risk of HCC. In addition, rs1800734 had an interaction with SNPs rs10849, rs3774343 and rs1540354, these SNP-SNP interactions also increased HCC risk, suggesting that *MLH1* polymorphisms play an important role in the development of HCC. However, we didn’t find any interaction between *MLH1* polymorphisms and family history of cancer, which may be due to the small sample size and low test efficiency.

HCC is a highly malignant tumor with a very poor prognosis [36–38]. Accumulated evidences showed that the clinical-pathological features were closely related to the prognosis of tumor, such as tumor size, tumor number, tumor stage, AFP level, lymph node metastasis, tumor thrombus, liver cirrhosis, vascular invasion and migration predicted poor prognosis for HCC [39–42]. This study clarified that rs1800734 was correlated with tumor size, tumor grade and AFP level, indicating that gene polymorphisms may cause carcinogenesis and different clinical-pathological features of patients [39, 42]. Furthermore, we found rs1800734, tumor number, tumor stage and lymph node metastasis were correlated with the prognosis of HCC in a COX regression analysis, and rs1800734 decreased the survival time of HCC patients significantly, suggesting that rs1800734 was a risk factor of HCC prognosis.

In addition, rs1800734 is a transcription factor binding site (TFBS), which can cause individual susceptibility difference by regulating MLH1 activity and the expression of downstream proteins [19, 22]. The relationship between rs1800734 and HCC prognosis may result from its regulation of the binding ability of transcription factors on MLH1, and then lead to expression change of MLH1 in HCC. However, the exact mechanism need further study to prove.

In summary, this is the first report of the relationship between *MLH1* polymorphisms with the risk and prognosis of HCC. We found that rs1800734 increased the risk of HCC and was a risk factor for poor prognosis of HCC, which is expected to become a new biomarker of HCC with poor prognosis.

## MATERIALS AND METHODS

### Ethics statement

The study was approved by the ethics committee of the First Affiliated Hospital of Guilin Medical University. All patients were informed about the aims of sample collection and signed the informed consent in accordance with the ethical guidelines of the hospital.

### Study population

1,036 new cases of HCC patients were collected as the case group from Department of Hepatobiliary Surgery, the First Affiliated Hospital of Guilin Medical University and the Guangxi Medical University between July 2009 and June 2015. These HCC patients were diagnosed as HCC by radiological or pathological methods and had not received chemotherapy and radiotherapy before the blood collection. 1,036 cases of non-HCC patients at the same time were selected as the control group from Department of Spinal Bone Marrow Surgery and Hand Trauma Surgery, the First Affiliated Hospital of Guilin Medical University and the Guangxi Medical University, with gender, age and nation matched.

### Blood sample collection and investigation

The investigation questionnaire was designed after consulting experts and conducted by trained investigators in a face-to-face way. The questionnaire included general information, such as past history, personal history, family history, smoking and alcohol intake, and clinical data, such as tumor size, number, staging, portal vein tumor thrombus (PVTT), hepatitis virus infection, AFP level, histological grade, and lymph node metastasis. 2 ml peripheral blood was collected from all patients for DNA extraction. The blood DNA was stored at −80°C.

### Follow up

475 cases of HCC patients underwent surgical resection between July 2010 and June 2015 were chosen in follow-up by telephone or outpatient review. Since the date of entering the group, they were follow-up every six months until June 2016. The survival time was counted from the first day after operation to the day when patients had metastasis, recurrence, death or the end of the follow-up. At the end of follow-up, 39 patients were lost and 436 patients had complete follow-up data.

The inclusion criteria for follow-up patients: (1) could undergo radical surgery after preoperative blood and imaging examination; (2) were confirmed as HCC by clinical pathology after radical surgery; (3) provided the blood sample; (4) had complete clinical and prognosis information; (5) signed the informed consent. The exclusion criteria for follow-up patients: (1) had not undergone radical surgery; (2) without complete clinical information; (3) refused to provide blood sample; (4) refused to sign the informed consent.

### Genotyping

Genotyping was conducted on Applied Biosystems 7500 Fast Real-Time PCR System (ABI, United States) by TaqMan MGB high throughput RT-PCR method and the results were analyzed on 7500 Fast System V1.4.0 SDS software. For quality control, 5% sample DNA was chosen to repeat the genotyping and the concordance rate was 100%. The genotyping was repeated once again when the sample couldn’t be genotyped, and the sample was abandoned if it couldn’t be genotyped in the repeat genotyping.

### Statistical analysis

All statistical analysis was conducted using SPSS 19.0 software. Hardy Weinberg equilibrium (HWE) test in the control group was performed using Haploview 4.2. Qualitative and quantitative data were analyzed by χ^2^ and *t* test, respectively. SNP-SNP interaction, gene-environment interaction, 95% confidence interval (CI) and odds ratio (OR) were analyzed by binary logistic regression model. The median survival time (MST) was calculated from the date of diagnosis to the date of death or the end of follow-up (June 2016). The overall survival curves were draw by Kaplan-Meier method and the differences between groups were analyzed using log-rank test. Multivariate COX regression was used for calculating hazard ratio (HR) and 95% CI. All tests were two-tailed and *P* < 0.05 was considered statistically significant.

## SUPPLEMENTARY MATERIALS FIGURE AND TABLES


