# Supplementary material for: Single nucleotide polymorphisms in MLH1 predict poor prognosis of hepatocellular carcinoma in a Chinese population
Source: Oncotarget. 2017 Apr 6;8(45):80039–49. doi: 10.18632/oncotarget.16899 (PMC5668119; doi:10.18632/oncotarget.16899)
Supplement: Supplementary file 1 [file oncotarget-08-80039-s001.pdf]

Single nucleotide polymorphisms in *MLH1* predict poor prognosis of hepatocellular carcinoma in a Chinese population

Supplementary Materials

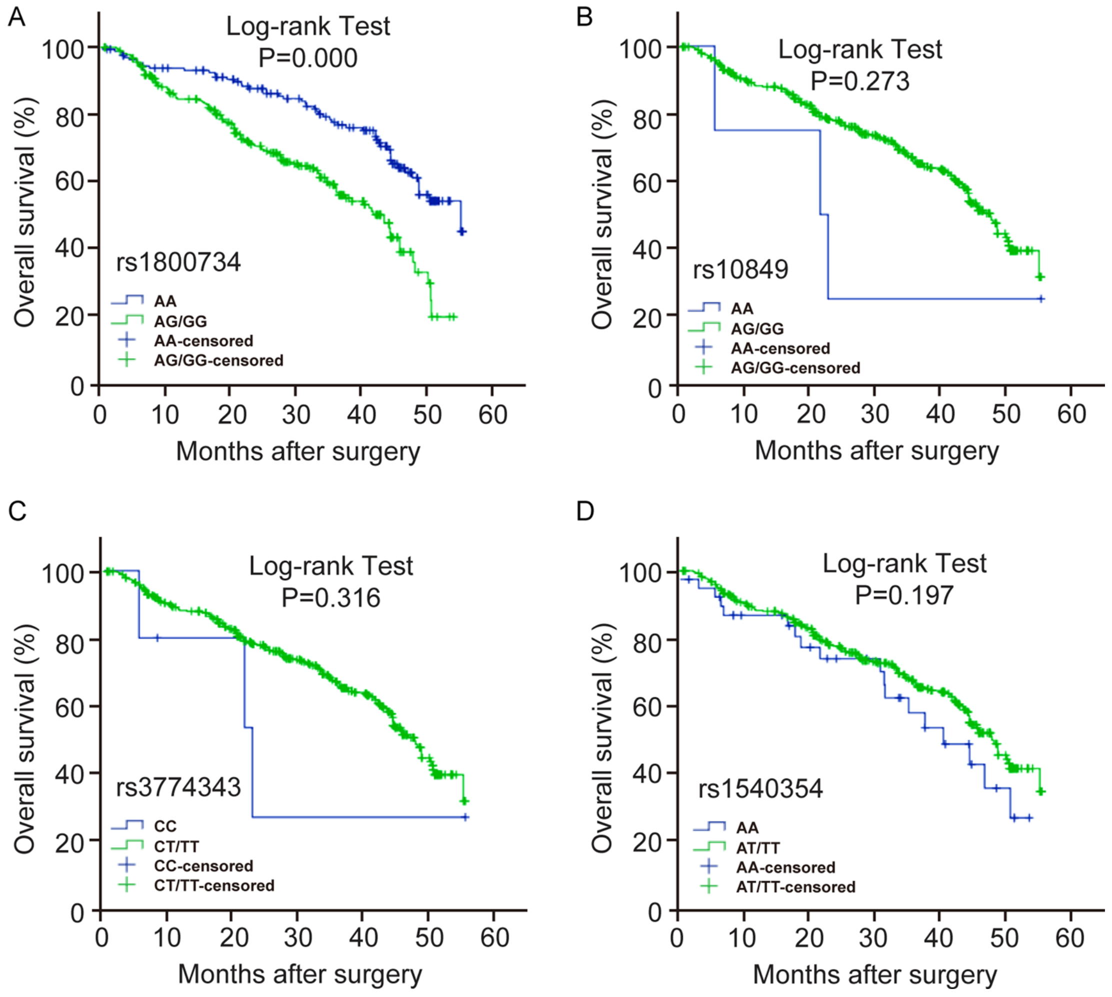

Supplementary Figure 1: Kaplan-Meier overall survival curves for HCC patients based on recessive genotypes of (**A**) rs1800734, (**B**) rs10849, (**C**) rs3774343, (**D**) rs1540354. P value is from the log-rank test.

Supplementary Table 1: Hardy-Weinberg equilibrium test results in controls

SNPs	Genotypes	Controls (n = 1036)		χ^2	*P*
		n	%		
rs1800734	AA	338	32.63	2.503	0.114
	AG	529	51.06		
	GG	169	16.31		
rs10849	AA	3	0.29	2.525	0.112
	AG	162	15.64		
	GG	871	84.07		
rs3774343	CC	2	0.19	1.151	0.283
	CT	125	12.06		
	TT	909	87.75		
rs1540354	AA	81	7.82	1.619	0.203
	AT	447	43.15		
	TT	508	49.03		

Supplementary Table 2: SNP-SNP interaction

Factors	*β*	*S.E.*	*Wald* χ^2	OR (95% CI)[a]	*P*
rs1800734 × rs10849	0.657	0.211	11.539	1.586 (1.142~4.373)	**0.000**
rs1800734 × rs3774343	0.745	0.196	13.756	1.763 (1.251~5.395)	**0.00**
rs1800734 × rs1540354	0.326	0.235	4.573	1.185 (1.026~3.614)	**0.023**
rs10849 × rs3774343	0.285	0.217	2.872	1.122 (0.927~3.265)	0.065
rs10849 × rs1540354	0.264	0.228	2.359	1.103 (0.757~2.748)	0.152
rs3774343 × rs1540354	0.241	0.239	2.221	1.087 (0.685~2.437)	0.226

[a]: OR (95% CI) adjusted by logistic regression for age, gender, nations, smoking, alcohol intake, HBV infection, and HCC family history. Bold values indicate significance.
